# Application of aiAtlas Version 1.2 to Simulate Variant Gene Function Restoration and Rescue Thresholds in Rare Diseases: Validation Case Study

**DOI:** 10.2196/88672

**Published:** 2026-09-22

**Authors:** Wayne Danter

**Affiliations:** 1 HumanQAI, Inc, #14-500 Sunnystone Road, London, ON, N5X 4R4, Canada, 1 2266781440

**Keywords:** mechanistic simulation, large-concept model, virtual cell lines, gene variant modeling, rare disease modeling, causal AI, computational cell biology, artificial intelligence, AI

## Abstract

**Background:**

Xeroderma pigmentosum group D (XPD), caused by ERCC2 gene dysfunction, leads to defective nucleotide excision repair and hypersensitivity to UV radiation. Robust experimental models for variant-level functional assessment remain limited.

**Objective:**

This study aimed to use aiAtlas version 1.2, a mechanistic large-concept simulation model, to evaluate the functional consequences of ERCC2 (XPD) variants and identify quantitative thresholds for functional rescue under graded gene function restoration.

**Methods:**

We simulated 136 virtual artificially induced pluripotent stem cell–derived cell lines spanning wild-type, single-mutation, multi-mutation, human tumor–derived, and gene fusion ERCC2 states. Twenty-five features encompassing DNA damage and repair, replication stress, pluripotency, and epigenetic remodeling were analyzed using nonparametric statistics with Bonferroni correction, Hodges-Lehmann estimates, and Cliff δ with bootstrap CIs. Graded ERCC2 restoration (0%‐100%) was simulated to evaluate rescue thresholds. Cross-validation, bagging, and bootstrap resampling tested robustness.

**Results:**

Simulations identified 3 nonlinear ERCC2 (XPD) rescue thresholds: approximately 20% to 30% (initial stabilization of repair), approximately 50% (near normalization), and more than 80% (full convergence). Reduced-function variants required substantially less restoration than strict loss-of-function variants to cross each threshold. Group comparisons across variant classes showed consistent differences in DNA repair, replication stress, and epigenetic remodeling features supported by effect size metrics and CIs.

**Conclusions:**

aiAtlas version 1.2 provides a simulation-based mechanistic framework for variant-level functional modeling in ERCC2 (XPD). The simulations identified quantitative thresholds that support mechanistic hypothesis generation regarding variant-dependent restoration of function. These findings provide a computational basis for future studies and require independent validation before clinical application.

## Introduction

Understanding how genetic alterations drive disease phenotypes at the cellular level remains one of the fundamental challenges in biomedical research. While induced pluripotent stem cells (iPSCs) and established human cell lines have provided powerful experimental systems, they remain constrained by cost, scalability, and an inability to fully capture the systemic consequences of complex genomic states [1,2]. These limitations are particularly important in the context of rare and ultrarare diseases, where patient material is often scarce [3], and in cancers, where multiple mutations and gene fusions often interact in unpredictable ways [4,5].

While computational models continue to evolve, many current approaches remain limited in their ability to reproduce multi-pathway interactions across cellular and system levels [6]. There remains an unmet need for a platform that can integrate genomic, epigenomic, and cellular features to produce more physiologically realistic and testable predictions.

We recently developed aiAtlas, a simulation-based platform built on large-concept model (LCM) logic and artificially iPSC (aiPSC) modeling designed to simulate cellular states and model phenotypic divergence arising from a broad array of genetic perturbations. The platform draws on concept-based representation and systems-biology modeling principles [6,7]. LCMs represent an extension of traditional fuzzy cognitive maps (FCMs). While FCMs are constrained to explicit networks of weighted causal relationships between user-specified concepts (nodes), LCMs operate in a higher-dimensional concept space that allows for abstraction, improved generalization, and the emergence of higher-order interactions beyond smaller, manually constrained causal networks. LCMs represent a powerful AI architecture that extends beyond basic causal linkages to capture the broader conceptual dynamics of complex systems, making LCM-based approaches well suited for large-scale mechanistic biological modeling.

In part 1 of this study, we evaluated the ability of aiAtlas to reliably distinguish wild-type (WT) aiPSCs from a diverse population of mutant cells, capturing major shifts in cell cycle regulation, DNA repair, epigenetic remodeling, pluripotency, and the hallmarks of cancers. In part 2, we extended this framework to clinically relevant subgroups, each representing a distinct biological context, including single gene mutations, multiple mutations, human tumor–derived cell lines, and gene fusions. By systematically comparing these 4 subgroups against WT aiPSCs, we aimed to determine whether aiAtlas could not only detect broad divergence but also resolve more granular, subgroup-specific phenotypes. The current study evaluated whether aiAtlas could reproducibly simulate variant-associated phenotypic divergence and identify mechanistically credible ERCC2 (XPD) rescue thresholds across multiple aiPSC-derived virtual cell cohorts.

To further illustrate a potential translational application, in part 3, we applied aiAtlas to xeroderma pigmentosum group D (XPD), a rare DNA repair disorder caused by ERCC2 loss of function. This case study highlights how aiAtlas can estimate model-derived functional restoration thresholds for both rescue and normalization, providing a quantitative framework for exploring gene editing and pharmacologic strategies.

## Methods

### aiAtlas Simulation Platform

All analyses were performed using aiAtlas, a high-resolution computational modeling framework that integrates LCM-based causal inference with aiPSC simulation libraries. The platform simulates cellular states by combining genetic, epigenetic, and cell-signaling features into interconnected causal networks. Each concept (node) represents a measurable biological process, and edges define directional and weighted causal relationships between nodes on a continuous scale from −1 to +1. Simulations were iteratively propagated until optimal early stopping states were reached. Early stopping was used as a regularization method to improve generalizability and minimize the potential for overfitting the data.

### Update Logic, Early Stopping, and Convergence Checks

State updates were synchronous and followed a bounded nonlinear function of the form *x_t_*_+1_ = *f*(*x_t_W*), with element-wise squashing to the [−1, +1] scale (implementation details are proprietary but were fixed across all runs). A run was considered converged when the per-iteration vector change fell below a fixed tolerance across consecutive iterations; otherwise, bounded oscillations were summarized by their stable moving average. Early stopping safeguards were applied uniformly to prevent spurious drift and maintain consistent generalization behavior across cohorts and restoration levels.

### ERCC2 (XPD) Case Study (Gene Therapy Threshold Simulation)

In part 3, ERCC2 function was modeled at 6 restoration levels (0%, 10%, 20%, 50%, 80%, and 100%) using aiCRISPRL editing logic [8], with outputs including DNA damage burden, DNA repair, gH2AX, checkpoint activation, and genome instability. Input relationships linked ERCC2 efficiency to nucleotide excision repair (NER) core, global genomic NER, and transcription-coupled NER repair pathways.

Post hoc analysis of threshold stability was performed using the ERCC2 (XPD) restoration trajectory to evaluate whether major functional transition regions remained preserved across neighboring restoration intervals rather than relying on single-point thresholds. Stability was inferred when recovery classifications maintained monotonic ordering and consistent transition region behavior across the restoration profile.

### Cell Line Cohorts

In part 1, we analyzed 136 aiPSC lines using the aiAtlas platform comparing 10 WT lines with 126 mutant lines. In part 2, the simulated aiPSC lines were grouped into 4 simulated cohorts for comparison against WT lines (n=10): single mutations (n=81), multiple mutations (n=20), human tumor–derived cell lines (n=10), and gene fusion lines (n=15). All simulations were run under identical baseline conditions, with differences arising only from input mutational profiles. All aiPSC WT and mutated lines were generated using the aiCRISPRL gene editing simulation technology [8].

### Feature Definitions

Twenty-five biological and output features were evaluated and grouped into categories: cell cycle regulation, apoptosis and autophagy, DNA damage and repair, epigenetic remodeling, pluripotency and self-renewal, oncogenic features, stress responses, etc. Each feature was scaled to the [−1, +1] range, where negative values represent reduced or dysregulated function relative to the WT aiPSC state. Supporting feature definitions and associated references are provided in Multimedia Appendix 1.

### Statistical Analysis

Pairwise group comparisons were performed between WT and all mutated groups or subgroups. The nonparametric Mann-Whitney *U* test was used to assess distributional differences (*P* values) [9]. Multiple-testing correction was applied across the 25 features jointly using Bonferroni adjustment (*P*<.002) [10]. This threshold was applied consistently in parts 1 and 2. The Hodges-Lehmann estimator (HLE) was reported as the median of all pairwise differences between groups [11], presented as a point estimate without CIs. The Cliff δ was reported as a nonparametric effect size with 95% CIs [12].

Overall significance was evaluated using a multi-criterion framework. For each of the 25 features tested, we applied Bonferroni correction (adjusted threshold *P*<.002) to control for familywise error. In addition, we calculated the HLE for median differences and Cliff δ for effect size with 95% CIs for reproducibility. While Bonferroni provides strict control of false positives, features with nonsignificant adjusted *P* values but large effect sizes and narrow CIs were considered biologically meaningful and are reported as such. This approach balances statistical conservatism with detection of consistent, reproducible effects.

Importantly, 95% CIs for the Cliff δ were estimated using bootstrap resampling within the aiHumanoid (version 11.9) framework, with approximately 10,000 stratified resamples. This approach can yield repeated interval widths across features when effect sizes approach their bounds, which is expected behavior and reflects the resampling distribution rather than a coding artifact.

### Internal Validation and Reproducibility

All simulations were repeated independently to confirm stability of outcomes. Internal quality control included convergence monitoring, 5-fold cross-validation, bootstrap resampling, system-wide error, and stability indexes. Together, these procedures assess internal consistency and robustness of simulated outputs rather than external biological validation. All reported analyses were performed using deterministic computational simulations within aiAtlas version 1.2 under the conditions described above. No experimental, animal, organoid, or prospective clinical data were generated as part of this study.

### Ethical Considerations

This study involved computer simulations exclusively. No human participants, patient data, or animal studies were used. Ethics approval was not required.

## Results

### Overview

We analyzed 136 aiPSC-derived cell lines using the aiAtlas platform comparing 10 WT lines with 126 mutant lines. Twenty-five biological and cellular features were evaluated using nonparametric methods. Group differences were tested using the Mann-Whitney *U* test.

In part 1, we compared all 126 mutated aiPSC-derived cell lines to 10 WT aiPSCs. In part 2, we divided the data to create 4 specific subgroups for comparison with the aiPSC WT virtual cells.

### Part 1: Overall Findings

Across cohorts, the largest and most consistent differences were observed in pathways linked to DNA damage responses, epigenetic regulation, cell cycle control, and pluripotency markers. Several comparisons approached near-complete separation between WT and mutant distributions, with adjusted δ values ranging from –0.99 to +0.99. In these cases, saturation corrections were applied to avoid artificial boundary effects in effect size estimation. In contrast, several pathways—including DNA NER core processes and some metabolic or differentiation measures—showed negligible or small effect sizes, indicating no reproducible differences between WT and mutant states. Summary statistics for WT vs all mutated lines are shown in Table 1.

**Table 1.** Artificially induced pluripotent stem cell wild-type vs all mutated lines (selected features).

Features	HLE^a^	Mann-Whitney *U* test *P* value	Cliff δ (±95% CI)
Apoptosis_Extrinsic pathway	−0.100	1.65E-06	−0.913 (0.253)
Apoptosis_Intrinsic pathway	−0.098	7.31E-05	−0.751 (0.409)
DNA_Damage_Burden and accumulation	−0.290	2.15E-12	−0.978 (0.130)
DNA_Damage_CPD^b^ and 6-4_PPs^c^	0.341	4.99E-05	0.773 (0.393)
G1/S_Transition	−0.800	3.33E-06	−0.871 (0.305)
G2/M_Transition	−0.427	2.88E-03	−0.568 (0.510)
Genome_Instability and CIN^d^	−0.336	3.89E-04	−0.676 (0.457)
Marker_Stability	−0.311	5.83E-04	−0.656 (0.468)
Hallmarks of cancer	−0.989	4.00E-06	−0.878 (0.297)

a HLE: Hodges-Lehmann estimator.

b CPD: cyclobutane pyrimidine dimer.

c PP: pyrimidine-pyrimidone photoproduct.

d CIN: chromosomal instability.

### Part 12: Interpretation

#### Group 1: aiPSC WT vs All Mutated Cell Lines

In the largest cohort, consistent negative shifts were detected in cellular stress, mitochondrial stress, reactive oxygen species (ROS), and oxidative stress, and DNA replication stress (δ≈–0.998, 95% CI ±0.04). DNA damage burden and epigenetic dysfunction similarly demonstrated large negative effects (δ≈–0.98 to –0.99). In contrast, ERCC2_A was strongly positive (δ≈+0.998). Pluripotency markers NANOG, OCT3/4, and SOX2 were moderately to strongly negative. By comparison, DNA NER core processes showed δ values close to zero with wide CIs, consistent with no meaningful difference from WT cell lines. Complete statistical results are provided in Multimedia Appendix 2.

#### Group 2: aiPSC WT vs Single-Mutation Cell Lines

Patterns were broadly consistent with those of group 1, with saturation-adjusted δ values again near 0.998 for cellular stress, DNA replication stress, epigenetic dysfunction, mitochondrial stress, ERCC2_Activity, and ROS and oxidative stress. Strong effect sizes (|δ|≈0.7‐0.85) were also identified for G2/M transition, gH2AX, and genome instability (chromosomal instability). However, several repair pathways, including DNA NER core and certain transition checkpoints, showed weak or no measurable differences, underscoring variability in single-mutant effects. Comparative results for single-mutation aiPSC lines are shown in Table 2.

**Table 2.** Artificially induced pluripotent stem cell wild-type cell lines vs single mutations. Full results for all 25 features can be found in Multimedia Appendix 2.

Features	HLE^a^	Mann-Whitney *U* test *P* value	Cliff δ (±95% CI)
Apoptosis_Extrinsic pathway	−0.100	2.81E-07	−0.998 (0.044)
Apoptosis_Intrinsic pathway	−0.099	3.03E-10	−0.958 (0.178)
DNA_Damage_Burden and accumulation	−0.268	1.87E-12	−0.993 (0.075)
DNA_Damage_CPD^b^ and 6-4_PPs^c^	0.357	2.59E-05	)0.820 (0.355)
G1/S_Transition	−0.800	1.25E-07	−0.998 (0.044)
G2/M_Transition	−0.476	1.68E-04	−0.733 (0.421)
Genome_Instability and CIN^d^	−0.343	1.68E-04	−0.733 (0.421)
Marker_Stability	−0.320	2.90E-05	−0.815 (0.359)
Hallmarks of cancer	−1.000	2.69E-07	−0.998 (0.044)

a HLE: Hodges-Lehmann estimator.

b CPD: cyclobutane pyrimidine dimer.

c PP: pyrimidine-pyrimidone photoproduct.

d CIN: chromosomal instability.

#### Group 3: aiPSC WT vs Multiple-Mutation Cell Lines

With smaller group sizes, δ estimates remained large but displayed greater variability. G1/S transition, DNA replication stress, epigenetic dysfunction, and mitochondrial stress again reached saturation correction levels (δ≈ ±0.99, 95% CI ±0.09). Genome instability and DNA damage burden also showed strong negative effects (δ≈–0.87). In contrast, several DNA repair end points and differentiation markers yielded low δ values with wide CIs, consistent with no significant difference. Again, HLE median differences were more variable. Detailed outcomes for multiple-mutation aiPSC lines are shown in Table 3.

Full results for all 25 features are shown in Multimedia Appendix 2.

**Table 3.** Artificially induced pluripotent stem cell wild-type cell lines vs multiple mutations.

Features	HLE^a^	Mann-Whitney *U* test *P* value	Cliff δ (±95% CI)
Apoptosis_Extrinsic pathway	−0.100	6.12E-06	−0.900 (0.270)
Apoptosis_Intrinsic pathway	−0.075	8.14E-02	−0.400 (0.568)
DNA_Damage_Burden and accumulation	−0.398	1.20E-05	−0.990 (0.087)
DNA_Damage_CPD^b^ and 6-4_PPs^c^	0.343	3.22E-03	0.650 (0.471)
G1/S_Transition	−0.821	9.67E-06	−0.990 (0.087)
G2/M_Transition	−0.478	3.68E-03	−0.640 (0.476)
Genome_Instability and CIN^d^	−0.446	2.24E-05	−0.870 (0.306)
Marker_Stability	−0.381	2.24E-05	−0.870 (0.306)
Hallmarks of cancer	−1.026	1.17E-05	−0.990 (0.087)

a HLE: Hodges-Lehmann estimator.

b CPD: cyclobutane pyrimidine dimer.

c PP: pyrimidine-pyrimidone photoproduct.

d CIN: chromosomal instability.

#### Group 4: aiPSC WT vs Human Tumor–Derived Cell Lines

In the smallest group, highly consistent effects persisted for DNA replication stress, epigenetic dysfunction, mitochondrial stress, and pluripotency markers (δ≈–0.98, 95% CI ±0.12). ERCC2_Activity was again strongly positive (δ≈+0.98). Broader measures of cell cycle control such as G1/S transition and apoptosis extrinsic pathway exhibited moderate to strong effect sizes (δ≈–0.8). However, several apoptosis-related measures and NER subpathways produced δ values near 0, indicating no reproducible differences. Comparisons between WT and human tumor–derived cell lines are summarized in Table 4.

The complete results for all 25 features are shown in Multimedia Appendix 2.

**Table 4.** Artificially induced pluripotent stem cell wild-type vs human cell lines.

Features	HLE^a^	Mann-Whitney *U* test *P* value	Cliff δ (±95% CI)
Apoptosis_Extrinsic pathway	−0.096	9.96E-04	−0.800 ( 0.372)
Apoptosis_Intrinsic pathway	0.041	4.66E-01	0.200 (0.607)
DNA_Damage_Burden and accumulation	−0.594	2.17E-05	−0.980 (0.123)
DNA_Damage_CPD^b^ and 6-4_PPs^c^	0.301	1.01E-01	0.440 (0.557)
G1/S_Transition	−0.832	1.74E-04	−0.980 (0.123)
G2/M_Transition	−0.663	1.38E-02	−0.640 (0.476)
Genome_Instability and CIN^d^	−1.430	2.92E-04	−0.880 (0.294)
Marker_Stability	−0.516	2.19E-02	−0.980 (0.123)
Hallmarks of cancer	−1.048	1.82E-04	−0.980 (0.123)

a HLE: Hodges-Lehmann estimator.

b CPD: cyclobutane pyrimidine dimer.

c PP: pyrimidine-pyrimidone photoproduct.

d CIN: chromosomal instability.

#### Group 5: aiPSC WT vs Cell Lines With Gene Fusions

Intermediate sample size results confirmed the overall trends. Saturation-adjusted δ values of ±0.987 were observed for cellular stress, DNA replication stress, epigenetic dysfunction, mitochondrial stress, ROS and oxidative stress, and ERCC2_Activity (95% CI ±0.10). DNA damage burden was strongly negative (δ≈–0.87), whereas DNA damage cyclobutane pyrimidine dimer and 6-4 pyrimidine-pyrimidone photoproducts were strongly positive (δ≈+0.91). Conversely, several baseline markers such as genome instability, DNA NER core, and general stress signals showed small or absent differences relative to WT cell lines. Findings for gene fusion cell lines are shown in Table 5.

Results for all 25 features are shown in Multimedia Appendix 2.

**Table 5.** Artificially induced pluripotent stem cell wild-type cell lines vs gene fusions.

Features	HLE^a^	Mann-Whitney *U* test *P* value	Cliff δ (±95% CI)
Apoptosis_Extrinsic pathway	0.000	2.04E-02	−0.533 (0.524)
Apoptosis_Intrinsic pathway	−0.021	1.27E-03	−0.733 (0.421)
DNA_Damage_Burden and accumulation	−0.238	7.71E-05	−0.867 (0.309)
DNA_Damage_CPD^b^ and 6-4_PPs^c^	0.179	2.75E-05	0.907 (0.261)
G1/S_Transition	0.003	7.33E-01	0.087 (0.617)
G2/M_Transition	0.159	5.31E-02	0.467 (0.548)
Genome_Instability and CIN^d^	0.028	9.27E-01	0.027 (0.620)
Marker_Stability	0.067	6.12E-02	0.453 (0.552)
Hallmarks of cancer	0.007	9.25E-01	0.027 (0.620)

a HLE: Hodges-Lehmann estimator.

b CPD: cyclobutane pyrimidine dimer.

c PP: pyrimidine-pyrimidone photoproduct.

d CIN: chromosomal instability.

#### Integrated Interpretation

Across all simulated cohorts, aiAtlas consistently identified coordinated divergence in stress response, DNA repair, replication stress, epigenetic remodeling, and pluripotency-associated pathways. In contrast, several baseline regulatory and NER core processes demonstrated relative stability across cohorts. Together, these findings support the ability of aiAtlas to distinguish both shared and subgroup-specific consequences of complex mutational states while preserving internally consistent pathway behavior.

Complete statistical results for all cohort comparisons are provided in Multimedia Appendix 2. Heat maps summarizing HLEs and Cliff δ effect sizes across all cohorts are provided in Multimedia Appendix 3.

### Part 3: ERCC2 (XPD) Therapeutic Rescue Thresholds

To explore a potential translational application, we modeled stepwise restoration of ERCC2 function in aiPSC-derived lines representing XPD (XPD/ERCC2). Six restoration levels were simulated (0%, 10%, 20%, 50%, 80%, and 100%) using the aiCRISPRL gene editing framework [8], with outputs including DNA damage accumulation, gH2AX, checkpoint activation, and genome instability.

The simulations revealed a nonlinear recovery trajectory:

- 0% restoration—severe loss of function, characterized by high DNA damage burden, increased gH2AX, and marked genome instability
- 10% restoration—minimal functional gain; apoptosis remained elevated, and DNA repair pathways were largely ineffective
- 20% to 30% restoration (initial rescue region)—first measurable improvement in DNA repair stability, with partial suppression of genome instability and checkpoint activity approaching WT levels
- Approximately 50% restoration (near-normalization region)—simulated near normalization of NER activity and DNA checkpoint regulation, with apoptosis burden reduced to baseline variance
- More than 80% restoration (full convergence region)—simulated functional convergence with WT profiles; diminishing incremental gains beyond 50%, consistent with nonlinear saturation behavior

Statistical analyses (Mann-Whitney *U* test with Bonferroni correction, HLEs, and Cliff δ with bootstrap CIs) supported the reproducibility and robustness of these transition regions across multiple simulations.

Post hoc perturbation analysis demonstrated preservation of the major recovery transition regions under modest variation in recovery values, supporting the robustness of the identified rescue, near-normalization, and convergence intervals.

**Figure 1.**
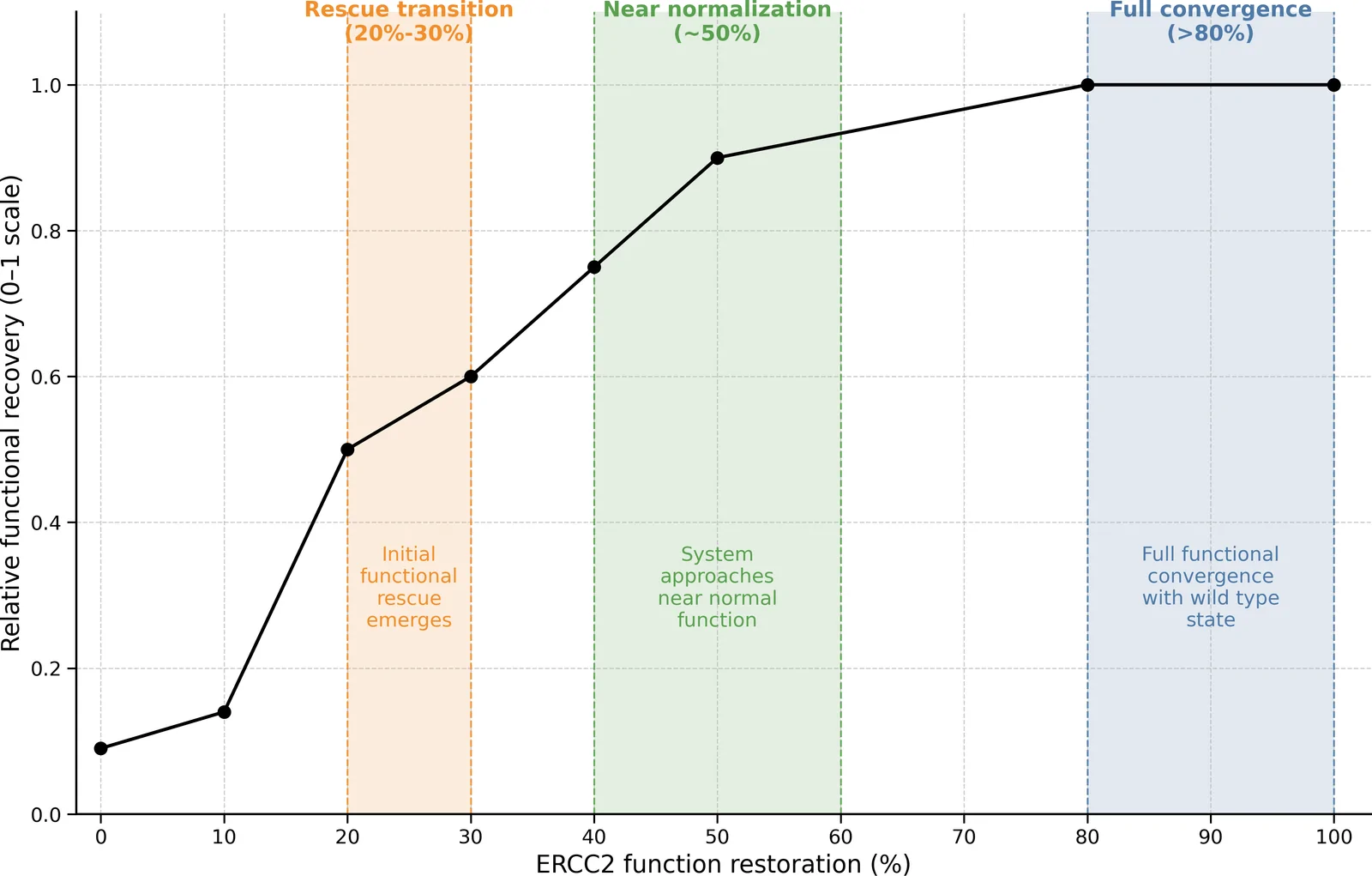
ERCC2 (xeroderma pigmentosum group D) functional recovery profile.

The major functional transition regions (approximately 20%-30%, approximately 50%, and >80%) remained consistent across all NER subpathways (NER-core, global genomic-NER, and transcription-coupled–NER), supporting a stable nonlinear recovery trajectory across restoration levels (Figure 1).

Simulated restoration of ERCC2 activity demonstrated a stable nonlinear recovery profile across increasing levels of functional restoration. Three broad transition regions defined key functional states: initial rescue (approximately 20%‐30%), near normalization (approximately 50%), and full convergence (>80%). The shaded bands in Figure 1 indicate the approximate threshold regions identified through post hoc threshold stability analysis. The monotonic recovery trajectory and preserved threshold ordering support the robustness of these functional transition regions across ERCC2 (XPD) restoration levels.

## Discussion

### Principal Findings

This simulation study demonstrates that aiAtlas can provide a mechanistic computational framework for modeling genetic alterations across diverse biological contexts in virtual aiPSC-derived cell lines. Across the simulated cohorts, aiAtlas identified broad divergence between WT and mutant states but also reliably resolved subgroup-specific phenotypic patterns and biological features that remained comparatively stable.

The most consistent simulation findings were large, internally consistent differences in pathways related to cellular stress, DNA replication, oxidative metabolism, and epigenetic remodeling. These results were observed across all subgroups, with Cliff δ effect sizes approaching complete separation (|δ|≈0.99) and narrow nonzero CIs even in smaller cohorts. Of note, ERCC2_Activity consistently showed a strong positive effect, supporting its role as an important determinant of the simulated cellular response to mutational stress. At the same time, some pathways—including DNA NER core processes, selected apoptosis measures, and certain differentiation markers—showed variable and small differences compared to WT cell lines. These stable features provide internal benchmarks, indicating that aiAtlas did not uniformly generate divergence but, instead, discriminated between simulated affected and unaffected cellular mutational states.

The subgroup analyses of the simulation data further supported these trends. Single mutations were often sufficient to generate systemic divergence, particularly in stress and epigenetic features, whereas multiple mutations amplified these effects and contributed to additional instability. Human tumor–derived virtual cell lines exhibited pronounced alterations in stress and pluripotency but retained some elements of overlap with WT cell lines, consistent with partial phenotypic conservation [4,5]. Gene fusion lines displayed an intermediate phenotype, with strong effects in selected DNA damage and repair outputs but relative stability in others, underscoring the pathway-selective nature of fusion-driven alterations [5].

These simulation results highlight both shared and subgroup-specific patterns. Common modeled pathway changes involved cell cycle dysregulation, increased genomic instability, and reduced pluripotency-associated features. Subgroup differences were most evident in apoptosis, DNA repair subpathways, and differentiation-related end points, where effect sizes were smaller or absent. Together, these findings suggest that aiAtlas can distinguish broad simulated systemic consequences of genetic perturbations from more context-dependent output changes.

This balance of divergence and stability supports the internal biological coherence of the aiAtlas version 1.2 simulations. Prior studies have shown that experimental iPSCs, while invaluable, face limitations in scalability, reproducibility, and capturing complex mutational interactions [1-3]. Computational models have attempted to address these gaps but are often unable to reproduce the complex multi-pathway interactions inherent in human biology [6,7]. By integrating LCM mechanistic logic with aiPSC simulations, aiAtlas provides a scalable computational approach for exploring these complex interactions [6-8].

The ERCC2 (XPD) simulations in part 3 illustrate how aiAtlas can be used to estimate model-derived functional restoration regions in rare DNA repair disorders. The simulated recovery profile was nonlinear and showed that partial correction within the approximately 20% to 30% region was associated with the earliest measurable improvement in DNA repair stability, whereas restoration levels centered around approximately 50% approached near normalization within the simulated system. These findings are broadly consistent with clinical reports of variable loss-of-function ERCC2 (XPD) alleles, where patients may exhibit attenuated or heterogeneous disease severity despite incomplete NER function [13-16]. Such variability across xeroderma pigmentosum, trichothiodystrophy, and xeroderma pigmentosum and trichothiodystrophy overlap syndromes is consistent with an association between residual ERCC2 gene function and mitigation of disease severity.

These simulation findings suggest several potential directions for future translational experiments: (1) gene editing studies may not need full correction to produce measurable biological improvement; (2) pharmacologic chaperones or small-molecule enhancers that boost residual ERCC2 activity into the modeled 20% to 50% range could be tested experimentally to detect any measurable DNA repair improvement; and (3) as computational new approach methodologies continue to evolve, model-derived restoration regions may also warrant evaluation as exploratory benchmarks for therapy development, provided they are supported by independent experimental validation.

Together, these findings illustrate how aiAtlas may serve as a quantitative mechanistic simulation framework for generating experimentally testable hypotheses about functional restoration in rare diseases.

### Limitations of the Study

This was a simulation study without matched wet laboratory validation; therefore, the quantitative thresholds should be interpreted as model-derived estimates pending experimental confirmation in iPSC and organoid systems. Accordingly, the reported rescue thresholds represent mechanistically credible, hypothesis-generating estimates rather than experimentally validated biological thresholds.

Metabolic, microenvironmental, and immune processes were only partially represented and may modulate effect sizes in specific contexts. Although internal robustness was assessed using 5-fold cross-validation, bagging, and bootstrap CIs, these procedures do not constitute biological validation. External benchmarking against independent experimental datasets remains future work. Finally, we did not implement a separate FCM baseline; the present LCM extends the FCM logic used in recent aiHumanoid projects and showed internally stable discrimination under the stated resampling procedures.

In conclusion, the analyses of the simulation data indicate that aiAtlas can consistently generate patterns of divergence and relative stability across multiple modeled genomic contexts. This combination of simulated divergence and stability supports the further evaluation of aiAtlas as a mechanistic hypothesis generation framework for rare disease modeling and cancer biology research. The scalable and biologically grounded simulation outputs support the continued development and experimental evaluation of whether aiAtlas can contribute to translational research and support regulatory innovation.

Importantly, in cases in which the target mutational profile has been defined but corresponding cellular models are unavailable, aiAtlas can generate custom virtual cell states that represent the specified genomic inputs and associated model-derived phenotypic outputs.

## Supplementary material

10.2196/88672 Multimedia Appendix 1 Artificially induced pluripotent stem cell line concepts.

10.2196/88672 Multimedia Appendix 2 Complete statistical results for all 25 biological features across all experimental cohorts, including Hodges-Lehmann estimates, Mann-Whitney *U* test results, Cliff δ effect sizes, and bootstrap-derived 95% CIs.

10.2196/88672 Multimedia Appendix 3 Heat maps for Hodges-Lehmann estimator and Cliff δ values for all 5 groupings across all 25 features.

## Abbreviations

aiPSC: artificially induced pluripotent stem cell
FCM: fuzzy cognitive map
HLE: Hodges-Lehmann estimator
iPSC: induced pluripotent stem cell
LCM: large-concept model
NER: nucleotide excision repair
ROS: reactive oxygen species
WT: wild type
XPD: xeroderma pigmentosum group D
