# Supplementary material for: Application of aiAtlas Version 1.2 to Simulate Variant Gene Function Restoration and Rescue Thresholds in Rare Diseases: Validation Case Study
Source: JMIR Bioinform Biotechnol. 2026 Sep 22;7:e88672. doi: 10.2196/88672 (PMC13597013; doi:10.2196/88672)
Supplement: Supplementary file 1 [file bioinform-v7-e88672-s001.docx]

**Multimedia Appendix 3. aiPSC Cell Line Concepts and Supporting References**

Supporting literature references and biological rationale for the principal aiAtlas v1.2 feature concepts used throughout the aiPSC simulation framework.

## Apoptosis - Extrinsic Pathway

1. Ashkenazi A, Dixit VM. Death receptors: signaling and modulation. Science. 1998;281(5381):1305-1308.
2. Fulda S, Debatin KM. Apoptosis signaling in tumor therapy. Ann N Y Acad Sci. 2004;1028:150-156. doi:10.1196/annals.1322.016. PMID:15650241.
3. Lavrik IN. Systems biology of death receptor networks: live and let die. Cell Death Dis.

2014;5(5):e1259.

## Apoptosis - Intrinsic Pathway

4. Youle RJ, Strasser A. The BCL-2 protein family: opposing activities that mediate cell death. Nat Rev Mol Cell Biol. 2008;9(1):47-59.
5. Czabotar PE, Lessene G, Strasser A, Adams JM. Control of apoptosis by the BCL-2 protein family: implications for physiology and therapy. Nat Rev Mol Cell Biol. 2014; 15(1):49-63. doi:10.1038/nrm3722.
6. Tait SWG, Green DR. Mitochondria and cell death: outer membrane permeabilization and beyond. Nat Rev Mol Cell Biol. 2010;11(9):621-632.

## Autophagy

7. Mizushima N, Levine B. Autophagy in mammalian development and differentiation. Nat Cell Biol. 2010;12(9):823-830.
8. Guan JL, Simon AK, Prescott M, Menendez JA, Liu F, Wang F, et al. Autophagy in stem cells. Autophagy. 2013;9(6):830-849. doi:10.4161/auto.24132. PMID:23486312.
9. Xu Y, Zhang Y, García-Cañaveras JC, et al. Chaperone-mediated autophagy regulates the pluripotency of embryonic stem cells. Science. 2020;369(6502):397-403.

## Cellular Stress

10. Mandal PK, Blanpain C, Rossi DJ. DNA damage response in adult stem cells: pathways and consequences. Nat Rev Mol Cell Biol. 2011;12(3):198-202. doi:10.1038/nrm3060. PMID:21304553.
11. Martello G, Smith A. The nature of embryonic stem cells. Annu Rev Cell Dev Biol.

2014;30:647-675.

## DNA Damage Burden / Accumulation

12. Momcilovic O, Knobloch L, Fornsaglio J, Varum S, Easley C, Schatten G. DNA damage responses in human induced pluripotent stem cells and embryonic stem cells. PLoS One. 2010;5(10):e13410. doi:10.1371/journal.pone.0013410. PMID:20976220.
13. Luo LZ, Gopalakrishna-Pillai S, Nay SL, Park SW, Bates SE, Zeng X, Iverson LE, O'Connor TR. DNA repair in human pluripotent stem cells is distinct from that in non-pluripotent human cells. PLoS One. 2012;7(3):e30541. doi:10.1371/journal.pone.0030541. PMID:22412831.
14. González F, Georgieva D, Vanoli F, Shi ZD, Stadtfeld M, Ludwig T, Jasin M, Huangfu D. Homologous recombination DNA repair genes play a critical role in reprogramming to a pluripotent state. Cell Rep. 2013;3(3):651-660. doi:10.1016/j.celrep.2013.02.005. PMID:23478019.

## DNA Damage - CPDs / 6-4 PPs

15. Pfeifer GP, You YH, Besaratinia A. Mutations induced by ultraviolet light. Mutat Res. 2005;571(1-2):19-31.
16. Rastogi RP, Richa, Kumar A, Tyagi MB, Sinha RP. Molecular mechanisms of ultraviolet radiation-induced DNA damage and repair. J Nucleic Acids. 2010;2010:592980.
17. Schärer OD. Nucleotide excision repair in eukaryotes. Cold Spring Harb Perspect Biol.

2013;5(10):a012609.

## DNA NER - Core Pathway

18. Marteijn JA, Lans H, Vermeulen W, Hoeijmakers JHJ. Understanding nucleotide excision repair and its roles in cancer and ageing. Nat Rev Mol Cell Biol. 2014;15(7):465-481.
19. Schärer OD. Nucleotide excision repair in eukaryotes. Cold Spring Harbor Perspectives in Biology. 2013;5(10):a012609.
20. Cleaver JE. Defective repair replication of DNA in xeroderma pigmentosum. Nature. 1968;218(5142):652-656. doi:10.1038/218652a0. PMID:5655953.

## DNA NER - Global Genome (GG-NER)

21. Sugasawa K, Ng JM, Masutani C, Iwai S, van der Spek PJ, Eker AP, Hanaoka F, Bootsma D, Hoeijmakers JH. Xeroderma pigmentosum group C protein complex is the initiator of global genome nucleotide excision repair. Mol Cell. 1998;2(2):223-232. doi:10.1016/S1097-2765(00)80132-X. PMID:9734359.
22. Riedl T, Hanaoka F, Egly JM. The comings and goings of nucleotide excision repair factors on damaged DNA. EMBO J. 2003;22(19):5293-5303.
23. Marteijn JA, Lans H, Vermeulen W, Hoeijmakers JHJ. Understanding nucleotide excision repair and its roles in cancer and ageing. Nat Rev Mol Cell Biol. 2014;15(7):465-481.

## DNA NER - Transcription Coupled (TC-NER)

24. Hanawalt PC, Spivak G. Transcription-coupled DNA repair: two decades of progress and surprises. Nat Rev Mol Cell Biol. 2008;9(12):958-970.
25. Fousteri M, Mullenders LHF. Transcription-coupled nucleotide excision repair in mammalian cells: molecular mechanisms and biological effects. Cell Res. 2008;18(1):73-84.

## DNA Replication Stress

26. Ahuja AK, Jodkowska K, Teloni F, et al. A short G1 phase imposes constitutive replication stress and fork remodelling in mouse embryonic stem cells. Nat Commun. 2016;7:10660. doi:10.1038/ncomms10660. PMID:26876348.
27. Desmarais JA, Hoffmann MJ, Bingham G, Gagou ME, Meuth M, Andrews PW. Human embryonic stem cells fail to activate CHK1 and commit to apoptosis in response to DNA replication stress. Stem Cells. 2012;30(7):1385-1393. doi:10.1002/stem.1117.

## Epigenetic Dysfunction

28. Nishino K, Toyoda M, Yamazaki-Inoue M, Fukawatase Y, Chikazawa E, Sakaguchi H, et al. DNA methylation dynamics in human induced pluripotent stem cells over time. PLoS Genet. 2011;7(5):e1002085. doi:10.1371/journal.pgen.1002085. PMID:21637780.
29. Nazor KL, Altun G, Lynch C, et al. Recurrent variations in DNA methylation in human pluripotent stem cells and their differentiated derivatives. Cell Stem Cell. 2012;10(5):620-634. doi:10.1016/j.stem.2012.02.013. PMID:22560082.
30. Lister R, Pelizzola M, Kida YS, et al. Hotspots of aberrant epigenomic reprogramming in human induced pluripotent stem cells. Nature. 2011;471(7336):68-73.

## ERCC2_Activity

31. Coin F, Marinoni JC, Rodolfo C, Fribourg S, Pedrini AM, Egly JM. Mutations in the XPD helicase gene result in XP and TTD phenotypes, preventing interaction between XPD and the p44 subunit of TFIIH. Nat Genet. 1998;20(2):184-188. doi:10.1038/2491. PMID:9771713.
32. Lehmann AR. The xeroderma pigmentosum group D (XPD) gene: one gene, two functions, three diseases. Genes Dev. 2001;15(1):15-23. doi:10.1101/gad.859501. PMID:11156600.
33. Fassihi H, Sethi M, Fawcett H, Wing J, Chandler N, Mohammed S, et al. Deep phenotyping of 89 xeroderma pigmentosum patients reveals unexpected heterogeneity dependent on the precise molecular defect. Proc Natl Acad Sci U S A. 2016;113(9):E1236-E1245. doi:10.1073/pnas.1519444113. PMID:26884178.

## G1/S Transition

34. Sherr CJ, Roberts JM. CDK inhibitors: positive and negative regulators of G1-phase progression. Genes Dev. 1999;13(12):1501-1512.
35. Neganova I, Lako M. G1 to S phase cell cycle transition in somatic and embryonic stem cells. J Anat. 2008;213(1):30-44. doi:10.1111/j.1469-7580.2008.00931.x. PMID:18638068.
36. Singh AM, Dalton S. The cell cycle and Myc intersect with mechanisms that regulate pluripotency and reprogramming. Cell Stem Cell. 2009;5(2):141-149. doi:10.1016/j.stem.2009.07.003. PMID:19664987.

## G2/M Transition

37. Nurse P. Universal control mechanism regulating onset of M-phase. Nature. 1990;344(6266):503-508.
38. Lindqvist A, Rodríguez-Bravo V, Medema RH. The decision to enter mitosis: feedback and redundancy in the mitotic entry network. J Cell Biol. 2009;185(2):193-202. doi:10.1083/jcb.200812045. PMID:19364923.

## Genome Instability / CIN

39. Negrini S, Gorgoulis VG, Halazonetis TD. Genomic instability-an evolving hallmark of cancer. Nat Rev Mol Cell Biol. 2010;11(3):220-228.
40. Bakhoum SF, Cantley LC. The multifaceted role of chromosomal instability in cancer and its microenvironment. Cell. 2018;174(6):1347-1360. doi:10.1016/j.cell.2018.08.027. PMID:30193109.
41. Sansregret L, Swanton C. The role of aneuploidy in cancer evolution. Cold Spring Harb Perspect Med. 2017;7(1):a028373. doi:10.1101/cshperspect.a028373. PMID:28049655.

gH2AX (γH2AX)

42. Rogakou EP, Pilch DR, Orr AH, Ivanova VS, Bonner WM. DNA double-stranded breaks induce histone H2AX phosphorylation on serine 139. J Biol Chem. 1998;273(10):5858-5868.
43. Bonner WM, Redon CE, Dickey JS, et al. γH2AX and cancer. Nat Rev Cancer. 2008;8(12): 957-967. doi:10.1038/nrc2523. PMID:19005492.
44. Mah LJ, El-Osta A, Karagiannis TC. γH2AX: a sensitive molecular marker of DNA damage and repair. Leukemia. 2010;24(4):679-686. doi:10.1038/leu.2010.6. PMID:20130602.

## DNA Hypermethylation

45. Nazor KL, Altun G, Lynch C, et al. Recurrent variations in DNA methylation in human pluripotent stem cells and their differentiated derivatives. Cell Stem Cell. 2012;10(5):620-634. doi:10.1016/j.stem.2012.02.013. PMID:22560082.

## DNA Hypomethylation / Demethylation

46. Lister R, Pelizzola M, Kida YS, et al. Hotspots of aberrant epigenomic reprogramming in human induced pluripotent stem cells. Nature. 2011;471(7336):68-73.
47. Ohi Y, Qin H, Hong C, Blouin L, Polo JM, Guo T, et al. Incomplete DNA methylation underlies a transcriptional memory of somatic cells in human iPS cells. Nat Cell Biol. 2011;13(5):541-549. doi:10.1038/ncb2239.

## Marker Stability

48. Koyanagi-Aoi M, Ohnuki M, Takahashi K, et al. Differentiation-defective phenotypes revealed by large-scale analyses of human pluripotent stem cells. Proc Natl Acad Sci U S A. 2013;110(51):20569-20574. doi:10.1073/pnas.1319061110. PMID:24259714.
49. Kim K, Doi A, Wen B, et al. Epigenetic memory in induced pluripotent stem cells. Nature. 2010;467(7313):285-290.
50. Bock C, Kiskinis E, Verstappen G, Gu H, Boulting G, Smith ZD, Ziller M, Croft GF, Amoroso MW, Oakley DH, Gnirke A, Eggan K, Meissner A. Reference maps of human ES and iPS cell variation enable high-throughput characterization of pluripotent cell lines. Cell. 2011;144(3):439-452. doi:10.1016/j.cell.2010.12.032. PMID:21295703.

## Mitochondrial Stress

51. Prigione A, Fauler B, Lurz R, Lehrach H, Adjaye J. The senescence-related mitochondrial/oxidative stress pathway is repressed in human induced pluripotent stem cells. Stem Cells. 2010;28(4):721-733. doi:10.1002/stem.404. PMID:20201066.
52. Folmes CDL, Dzeja PP, Nelson TJ, Terzic A. Metabolic plasticity in stem cell homeostasis and differentiation. Cell Stem Cell. 2012;11(5): 596-606.

## Nanog

53. Chambers I, Colby D, Robertson M, Nichols J, Lee S, Tweedie S, Smith A. Functional expression cloning of Nanog, a pluripotency sustaining factor in embryonic stem cells. Cell. 2003;113(5):643-655. doi:10.1016/S0092-8674(03)00392-1. PMID:12787505.
54. Silva J, Smith A. Capturing pluripotency. Cell. 2008;132(4):532-536. doi:10.1016/j.cell.2008.02.006. PMID:18295569.

OCT3/4 (POU5F1)

55. Niwa H, Miyazaki J, Smith AG. Quantitative expression of Oct-3/4 defines differentiation, dedifferentiation or self-renewal of ES cells. Nat Genet. 2000;24(4):372-376. doi:10.1038/74199. PMID:10742100.

## SOX2

56. Avilion AA, Nicolis SK, Pevny LH, Perez L, Vivian N, Lovell-Badge R. Multipotent cell lineages in early mouse development depend on SOX2 function. Genes Dev. 2003;17(1):126-140. doi:10.1101/gad.224503. PMID:12514105.
57. Masui S, Nakatake Y, Toyooka Y, Shimosato D, Yagi R, Takahashi K, et al. Pluripotency governed by Sox2 via regulation of Oct3/4 expression in mouse embryonic stem cells. Nat Cell Biol. 2007;9(6):625-635.

## ROS / Oxidative Stress

58. Armstrong L, Tilgner K, Saretzki G, Atkinson SP, Stojkovic M, Moreno R, Przyborski S, Lako M. Human induced pluripotent stem cell lines show stress defense mechanisms and mitochondrial regulation similar to those of human embryonic stem cells. Stem Cells. 2010;28(4):661-673. PMID:20073085.
59. Saretzki G, Armstrong L, Leake A, Lako M, von Zglinicki T. Stress defense in murine embryonic stem cells is superior to that of various differentiated murine cells. Stem Cells. 2004;22(6):962-971. doi:10.1634/stemcells.22-6-962. PMID:15536187.

## The Hallmarks of Cancer

60. Hanahan D, Weinberg RA. The hallmarks of cancer. Cell. 2000;100(1):57-70.
61. Hanahan D. Hallmarks of cancer: new dimensions. Cancer Discov. 2022;12(1):31-46.
