# Supplementary material for: Application of aiAtlas Version 1.2 to Simulate Variant Gene Function Restoration and Rescue Thresholds in Rare Diseases: Validation Case Study
Source: JMIR Bioinform Biotechnol. 2026 Sep 22;7:e88672. doi: 10.2196/88672 (PMC13597013; doi:10.2196/88672)
Supplement: Supplementary file 2 [file bioinform-v7-e88672-s002.docx]

Multimedia Appendix 1

Complete Statistical Results Across Experimental Cohorts

Complete statistical results for all 25 biological features across all experimental cohorts, including Hodges–Lehmann estimates, Mann–Whitney U test results, Cliff’s delta effect sizes, and bootstrap-derived 95% confidence intervals.

| Table S1a. aiPSC Wild Type vs All Mutated Cell Lines |  |  |  |  |
| --- | --- | --- | --- | --- |
| Feature | HLE | Mann-Whitney  p-value | Cliff's delta | ±95% CI |
| Apoptosis_Extrinsic pathway | -0.100 | 1.65E-06 | -0.913 | 0.253 |
| Apoptosis_Intrinsic pathway | -0.098 | 7.31E-05 | -0.751 | 0.409 |
| Autophagy | 0.485 | 4.69E-04 | 0.667 | 0.462 |
| Cellular stress | -0.016 | 1.52E-07 | -0.998 | 0.039 |
| DNA_Damage_Burden/Accumulation | -0.290 | 2.15E-12 | -0.978 | 0.130 |
| DNA_Damage_CPD/6-4_PPs | 0.341 | 4.99E-05 | 0.773 | 0.393 |
| DNA_NER_Core | 0.005 | 9.30E-01 | 0.017 | 0.620 |
| DNA_NER-GG | 0.572 | 9.19E-04 | 0.632 | 0.480 |
| DNA_NER-TC | 0.579 | 4.01E-04 | 0.675 | 0.458 |
| DNA_Replication Stress | -0.261 | 1.53E-07 | -0.998 | 0.039 |
| Epigenetic Dysfunction | -0.291 | 1.53E-07 | -0.998 | 0.039 |
| ERCC2_Activity | 1.888 | 1.52E-07 | 0.998 | 0.039 |
| G1S_Transition | -0.800 | 3.33E-06 | -0.871 | 0.305 |
| G2M_Transition | -0.427 | 2.88E-03 | -0.568 | 0.510 |
| Genome_Instability/CIN | -0.336 | 3.89E-04 | -0.676 | 0.457 |
| gH2AX | -0.431 | 9.19E-04 | -0.632 | 0.480 |
| HypermethylationDNA | -0.483 | 2.00E-05 | -0.813 | 0.361 |
| Hypomethylation/DemethylationDNA | -0.546 | 6.59E-06 | -0.859 | 0.318 |
| Marker_Stability | -0.311 | 5.83E-04 | -0.656 | 0.468 |
| Mitochondrial_Stress | -0.033 | 1.52E-07 | -0.998 | 0.039 |
| Nanog | -0.808 | 4.81E-06 | -0.854 | 0.323 |
| OCT_3/4 | -0.928 | 7.26E-06 | -0.838 | 0.338 |
| ROS/OxidativeStress | -0.029 | 2.43E-08 | -0.998 | 0.039 |
| Sox2 | -0.740 | 3.29E-06 | -0.841 | 0.335 |
| The Hallmarks of Cancer | -0.989 | 4.00E-06 | -0.878 | 0.297 |

Table S2a: aiPSC Wild Type vs Single Mutation Cell Lines

| Feature | HLE | Mann-  Whitney p-value | Cliff's delta | ±95% CI |
| --- | --- | --- | --- | --- |
| Apoptosis_Extrinsic pathway | -0.100 | 1.65E-06 | -0.913 | 0.253 |
| Apoptosis_Intrinsic pathway | -0.098 | 7.31E-05 | -0.751 | 0.409 |
| Autophagy | 0.485 | 4.69E-04 | 0.667 | 0.462 |
| Cellular stress | -0.016 | 1.52E-07 | -0.998 | 0.039 |
| DNA_Damage_Burden/Accumulation | -0.290 | 2.15E-12 | -0.978 | 0.130 |
| DNA_Damage_CPD/6-4_PPs | 0.341 | 4.99E-05 | 0.773 | 0.393 |
| DNA_NER_Core | 0.005 | 9.30E-01 | 0.017 | 0.620 |
| DNA_NER-GG | 0.572 | 9.19E-04 | 0.632 | 0.480 |
| DNA_NER-TC | 0.579 | 4.01E-04 | 0.675 | 0.458 |
| DNA_Replication Stress | -0.261 | 1.53E-07 | -0.998 | 0.039 |
| Epigenetic Dysfunction | -0.291 | 1.53E-07 | -0.998 | 0.039 |
| ERCC2_Activity | 1.888 | 1.52E-07 | 0.998 | 0.039 |
| G1S_Transition | -0.800 | 3.33E-06 | -0.871 | 0.305 |
| G2M_Transition | -0.427 | 2.88E-03 | -0.568 | 0.510 |
| Genome_Instability/CIN | -0.336 | 3.89E-04 | -0.676 | 0.457 |
| gH2AX | -0.431 | 9.19E-04 | -0.632 | 0.480 |
| HypermethylationDNA | -0.483 | 2.00E-05 | -0.813 | 0.361 |
| Hypomethylation/DemethylationDNA | -0.546 | 6.59E-06 | -0.859 | 0.318 |
| Marker_Stability | -0.311 | 5.83E-04 | -0.656 | 0.468 |
| Mitochondrial_Stress | -0.033 | 1.52E-07 | -0.998 | 0.039 |
| Nanog | -0.808 | 4.81E-06 | -0.854 | 0.323 |
| OCT_3/4 | -0.928 | 7.26E-06 | -0.838 | 0.338 |
| ROS/OxidativeStress | -0.029 | 2.43E-08 | -0.998 | 0.039 |
| Sox2 | -0.740 | 3.29E-06 | -0.841 | 0.335 |
| The Hallmarks of Cancer | -0.989 | 4.00E-06 | -0.878 | 0.297 |

| Table S3a: aiPSC Wild Type vs Multiple Mutation Cell Lines |  |  |  |  |
| --- | --- | --- | --- | --- |
|  |  |  |  |  |
| Feature | HLE | Mann-  Whitney p-value | Cliff's delta | ±95%  CI |
| Apoptosis_Extrinsic pathway | -0.100 | 6.12E-06 | -0.900 | 0.270 |
| Apoptosis_Intrinsic pathway | -0.075 | 8.14E-02 | -0.400 | 0.568 |
| Autophagy | 0.545 | 7.63E-04 | 0.770 | 0.395 |
| Cellular stress | -0.023 | 1.11E-05 | -0.990 | 0.087 |
| DNA_Damage_Burden/Accumulation | -0.398 | 1.20E-05 | -0.990 | 0.087 |
| DNA_Damage_CPD/6-4_PPs | 0.343 | 3.22E-03 | 0.650 | 0.471 |
| DNA_NER_Core | 0.359 | 3.92E-01 | 0.200 | 0.607 |
| DNA_NER-GG | 0.615 | 2.69E-02 | 0.500 | 0.537 |
| DNA_NER-TC | 0.675 | 4.68E-04 | 0.800 | 0.372 |
| DNA_Replication Stress | -0.277 | 1.20E-05 | -0.990 | 0.087 |
| Epigenetic Dysfunction | -0.312 | 1.20E-05 | -0.990 | 0.087 |
| ERCC2_Activity | 1.905 | 8.19E-06 | 0.990 | 0.087 |
| G1S_Transition | -0.821 | 9.67E-06 | -0.990 | 0.087 |
| G2M_Transition | -0.478 | 3.68E-03 | -0.640 | 0.476 |
| Genome_Instability/CIN | -0.446 | 2.24E-05 | -0.870 | 0.306 |
| gH2AX | -0.550 | 2.44E-04 | -0.780 | 0.388 |
| HypermethylationDNA | -0.518 | 1.33E-07 | -0.990 | 0.087 |
| Hypomethylation/DemethylationDNA | -0.677 | 5.83E-04 | -0.740 | 0.417 |
| Marker_Stability | -0.381 | 2.24E-05 | -0.870 | 0.306 |
| Mitochondrial_Stress | -0.038 | 8.19E-06 | -0.990 | 0.087 |
| Nanog | -0.867 | 9.74E-06 | -0.990 | 0.087 |
| OCT_3/4 | -0.932 | 8.10E-06 | -0.990 | 0.087 |
| ROS/OxidativeStress | -0.029 | 5.37E-06 | -0.990 | 0.087 |
| Sox2 | -0.793 | 3.88E-06 | -0.990 | 0.087 |
| The Hallmarks of Cancer | -1.026 | 1.17E-05 | -0.990 | 0.087 |

| Table S4a: aiPSC Wild Type vs Human Tumor–Derived Cell Lines |  |  |  |  |  |
| --- | --- | --- | --- | --- | --- |
|  |  |  |  |  |  |
| Feature |  | HLE | Mann-  Whitney p-value | Cliff's delta | ±95%  CI |
| Apoptosis_Extrinsic pathway |  | -0.096 | 9.96E-04 | -0.800 | 0.372 |
| Apoptosis_Intrinsic pathway |  | 0.041 | 4.66E-01 | 0.200 | 0.607 |
| Autophagy |  | 0.633 | 1.75E-02 | 0.620 | 0.486 |
| Cellular stress |  | -0.121 | 1.49E-04 | -0.980 | 0.123 |
| DNA_Damage_Burden/Accumulation |  | -0.594 | 2.17E-05 | -0.980 | 0.123 |
| DNA_Damage_CPD/6-4_PPs |  | 0.301 | 1.01E-01 | 0.440 | 0.557 |
| DNA_NER_Core |  | 0.818 | 1.36E-01 | 0.400 | 0.568 |
| DNA_NER-GG |  | 0.632 | 1.38E-01 | 0.400 | 0.568 |
| DNA_NER-TC |  | 0.719 | 1.37E-01 | 0.400 | 0.568 |
| DNA_Replication Stress |  | -0.559 | 1.82E-04 | -0.980 | 0.123 |
| Epigenetic Dysfunction |  | -0.462 | 1.82E-04 | -0.980 | 0.123 |
| ERCC2_Activity |  | 1.960 | 6.34E-05 | 0.980 | 0.123 |
| G1S_Transition |  | -0.832 | 1.74E-04 | -0.980 | 0.123 |
| G2M_Transition |  | -0.663 | 1.38E-02 | -0.640 | 0.476 |
| Genome_Instability/CIN |  | -1.430 | 2.92E-04 | -0.880 | 0.294 |
| gH2AX |  | -0.080 | 5.65E-01 | -0.160 | 0.612 |
| HypermethylationDNA |  | -0.508 | 1.82E-04 | -0.980 | 0.123 |
| Hypomethylation/DemethylationDNA |  | -0.616 | 1.82E-04 | -0.980 | 0.123 |
| Marker_Stability |  | -0.516 | 2.19E-02 | -0.980 | 0.123 |
| Mitochondrial_Stress |  | -0.054 | 6.39E-05 | -0.980 | 0.123 |
| Nanog |  | -0.932 | 1.62E-04 | -0.980 | 0.123 |
| OCT_3/4 |  | -0.933 | 1.80E-04 | -0.980 | 0.123 |
| ROS/OxidativeStress |  | -0.034 | 6.34E-05 | -0.980 | 0.123 |
| Sox2 |  | -0.862 | 1.62E-04 | -0.980 | 0.123 |
| The Hallmarks of Cancer |  | -1.048 | 1.82E-04 | -0.980 | 0.123 |

Table S5a: aiPSC Wild Type vs Gene Fusion Cell Lines

| Feature | HLE | Mann-  Whitney p-value | Cliff's delta | ±95%  CI |
| --- | --- | --- | --- | --- |
| Apoptosis_Extrinsic pathway | 0.000 | 2.04E-02 | -0.533 | 0.524 |
| Apoptosis_Intrinsic pathway | -0.021 | 1.27E-03 | -0.733 | 0.421 |
| Autophagy | -0.240 | 5.26E-02 | -0.467 | 0.548 |
| Cellular stress | -0.011 | 2.56E-05 | -0.987 | 0.100 |
| DNA_Damage_Burden/Accumulation | -0.238 | 7.71E-05 | -0.867 | 0.309 |
| DNA_Damage_CPD/6-4_PPs | 0.179 | 2.75E-05 | 0.907 | 0.261 |
| DNA_NER_Core | 0.061 | 9.98E-02 | 0.400 | 0.568 |
| DNA_NER-GG | 0.088 | 6.89E-02 | 0.440 | 0.557 |
| DNA_NER-TC | 0.051 | 9.97E-02 | 0.400 | 0.568 |
| DNA_Replication Stress | -0.084 | 3.58E-05 | -0.987 | 0.100 |
| Epigenetic Dysfunction | -0.086 | 3.58E-05 | -0.987 | 0.100 |
| ERCC2_Activity | 1.469 | 1.95E-05 | 0.987 | 0.100 |
| G1S_Transition | 0.003 | 7.33E-01 | 0.087 | 0.617 |
| G2M_Transition | 0.159 | 5.31E-02 | 0.467 | 0.548 |
| Genome_Instability/CIN | 0.028 | 9.27E-01 | 0.027 | 0.620 |
| gH2AX | 0.094 | 1.92E-01 | 0.320 | 0.587 |
| HypermethylationDNA | 0.193 | 2.20E-02 | 0.547 | 0.519 |
| Hypomethylation/DemethylationDNA | -0.018 | 4.21E-01 | -0.200 | 0.607 |
| Marker_Stability | 0.067 | 6.12E-02 | 0.453 | 0.552 |
| Mitochondrial_Stress | -0.011 | 1.95E-05 | -0.987 | 0.100 |
| Nanog | 0.043 | 3.60E-01 | 0.227 | 0.604 |
| OCT_3/4 | 0.086 | 1.41E-01 | 0.360 | 0.578 |
| ROS/OxidativeStress | -0.028 | 1.85E-05 | -0.987 | 0.100 |
| Sox2 | 0.076 | 1.74E-01 | 0.333 | 0.584 |
| The Hallmarks of Cancer | 0.007 | 9.25E-01 | 0.027 | 0.620 |
