# Supplementary material for: Application of aiAtlas Version 1.2 to Simulate Variant Gene Function Restoration and Rescue Thresholds in Rare Diseases: Validation Case Study
Source: JMIR Bioinform Biotechnol. 2026 Sep 22;7:e88672. doi: 10.2196/88672 (PMC13597013; doi:10.2196/88672)
Supplement: Supplementary file 3 [file bioinform-v7-e88672-s003.docx]

Multimedia Appendix 2.

Heat Maps of HLE and Cliff’s Delta Across Experimental Cohorts

Heat maps summarizing Hodges–Lehmann estimates and Cliff’s delta effect sizes across all wild-type and mutant cohort comparisons.

Figure S1: Heat Maps for HLE and Cliff’s delta values for all 5 groupings across all 25 Features

Figure S1: Legend-Color gradients represent relative magnitude and direction of effect sizes across 25 biological features for all five wild-type versus mutant cohort comparisons. Negative values (blue) indicate reduced relative activity compared with WT aiPSCs, whereas positive values (red) indicate increased relative activity. Large coordinated shifts were observed across stress-response, epigenetic-remodeling, pluripotency-associated, and genome-instability pathways.
